# Validation of a Smartphone-Based Approach to In Situ Cognitive Fatigue Assessment

**DOI:** 10.2196/mhealth.6333

**Published:** 2017-08-17

**Authors:** Edward Price, George Moore, Leo Galway, Mark Linden

**Affiliations:** ^1^ Computer Science Research Institute School of Computing Ulster University Newtownabbey United Kingdom; ^2^ School of Nursing and Midwifery Queen's University Belfast Belfast United Kingdom

**Keywords:** mental fatigue, fatigue, acquired brain injury, cognitive tests, assistive technology, smartphone

## Abstract

**Background:**

Acquired Brain Injuries (ABIs) can result in multiple detrimental cognitive effects, such as reduced memory capability, concentration, and planning. These effects can lead to cognitive fatigue, which can exacerbate the symptoms of ABIs and hinder management and recovery. Assessing cognitive fatigue is difficult due to the largely subjective nature of the condition and existing assessment approaches. Traditional methods of assessment use self-assessment questionnaires delivered in a medical setting, but recent work has attempted to employ more objective cognitive tests as a way of evaluating cognitive fatigue. However, these tests are still predominantly delivered within a medical environment, limiting their utility and efficacy.

**Objective:**

The aim of this research was to investigate how cognitive fatigue can be accurately assessed in situ, during the quotidian activities of life. It was hypothesized that this assessment could be achieved through the use of mobile assistive technology to assess working memory, sustained attention, information processing speed, reaction time, and cognitive throughput.

**Methods:**

The study used a bespoke smartphone app to track daily cognitive performance, in order to assess potential levels of cognitive fatigue. Twenty-one participants with no prior reported brain injuries took place in a two-week study, resulting in 81 individual testing instances being collected. The smartphone app delivered three cognitive tests on a daily basis: (1) Spatial Span to measure visuospatial working memory; (2) Psychomotor Vigilance Task (PVT) to measure sustained attention, information processing speed, and reaction time; and (3) a Mental Arithmetic Test to measure cognitive throughput. A smartphone-optimized version of the Mental Fatigue Scale (MFS) self-assessment questionnaire was used as a baseline to assess the validity of the three cognitive tests, as the questionnaire has already been validated in multiple peer-reviewed studies.

**Results:**

The most highly correlated results were from the PVT, which showed a positive correlation with those from the prevalidated MFS, measuring 0.342 (*P*<.008). Scores from the cognitive tests were entered into a regression model and showed that only reaction time in the PVT was a significant predictor of fatigue (*P*=.016, F=2.682, 95% CI 9.0-84.2). Higher scores on the MFS were related to increases in reaction time during our mobile variant of the PVT.

**Conclusions:**

The results show that the PVT mobile cognitive test developed for this study could be used as a valid and reliable method for measuring cognitive fatigue in situ. This test would remove the subjectivity associated with established self-assessment approaches and the need for assessments to be performed in a medical setting. Based on our findings, future work could explore delivering a small set of tests with increased duration to further improve measurement reliability. Moreover, as the smartphone assessment tool can be used as part of everyday life, additional sources of data relating to physiological, psychological, and environmental context could be included within the analysis to improve the nature and precision of the assessment process.

## Introduction

Cognitive fatigue can be caused by multiple different conditions, including Acquired Brain Injuries (ABIs) [1], Parkinson’s disease [2], stroke [3], heart failure [3], and many more. For those suffering from an ABI, cognitive fatigue can be triggered by carrying out simple everyday tasks, and is often much more prevalent [1]. Correct, timely, and accurate information is important to inform long-term management and rehabilitation from cognitive fatigue. Correspondingly, one of the primary challenges related to cognitive fatigue and its associated assessment is the subjective nature of the condition, coupled with a lack of identifiable biological markers. Consequently, there is a clear lack of technology-based approaches to assist with assessment and recovery management. Diagnosis and assessment typically relies upon assessment conducted within a clinical environment, meaning that incidents of cognitive fatigue can potentially go unrecognized, as they are most likely to happen when no professional or clinical supervision is present.

### Traditional Fatigue Assessment Approaches

Evaluation of cognitive fatigue has traditionally been performed using self-assessment in the form of easy-to-comprehend questionnaires that are often delivered within a clinical setting [4]. Accordingly, a number of self-assessment questionnaires have been designed to specifically assess cognitive ability and its relationship to fatigue, including the Mental Fatigue Scale (MFS) [5], Fatigue Severity Scale (FSS) [6], and Visual Analogue Scale for Fatigue (VAS-F) [7]. All of these scales use a visual analogue representation or targeted questions to aid a participant in self-evaluation. Upon completion, a clinician must then calculate the resulting score to categorize the level of cognitive fatigue. The MFS is a multidimensional questionnaire containing 15 questions developed by Johansson and Rönnbäck [8] (adapted from Rödholm et al [9]) which utilizes a range of questions that cover the most common symptoms occurring after an ABI [10,11]. The questions concern fatigue in general, lack of initiative, mental fatigue, mental recovery, concentration difficulties, memory problems, slowness of thinking, sensitivity to stress, increased tendency to become emotional, irritability, sensitivity to light and noise, and decreased or increased duration of sleep (as well as 24-hour variations). A resulting rating is calculated based on intensity, frequency, and duration of cognitive fatigue. The scale has been clinically evaluated and has adequate internal consistency with a Cronbach alpha of 0.94 [12]. Similarly, the FSS is a 9-item questionnaire that assesses the effect of general fatigue on daily living, in which each item is rated on a 7-point Likert scale [6]. Originally designed to assess general fatigue in patients with multiple sclerosis and systemic lupus erythematosus, the FSS has been successfully adapted to the measurement of fatigue in relation to other conditions [13]. By contrast, the VAS-F developed by Lee et al [4] employs an 18-item visual analogue scale to allow participants to determine their own rating regarding each statement in the scale. However, of these self-assessment questionnaires, the MFS is the only one that measures cognitive fatigue irrespective of neurological conditions [14].

### Computerized Fatigue Assessment Approaches

More recently, research into computerized cognitive fatigue assessment has sought to adopt cognitive testing methods by either adapting paper-based methods or repurposing cognitive testing batteries [15-17]. While cognitive testing methods have traditionally been used to determine relative cognitive ability, they have also been shown to indicate discrepancies in performance in relation to cognitive fatigue [16,18]. Van Dongen et al [19] defined three computerized methods for assessing fatigue: (1) a Mental Arithmetic Test to assess cognitive throughput; (2) the Psychomotor Vigilance Task (PVT) to assess reaction time; and (3) a Digit-Symbol Coding test to assess working memory. This study focused on measuring cognitive performance after periods of restricted sleep, with results indicating that even a relatively small amount of sleep restriction can seriously impair cognitive performance on everyday tasks [19].

Originally designed for a static desktop computer-based evaluation, PVT has since been modified for use on a mobile-based platform (to improve the utility of on-the-go assessment [16,20]) and has been shown to be an accurate predictor of vigilance due to fatigue and sleep loss [21,22], which is a direct predictor of cognitive fatigue. A shorter 3-minute version of PVT has been shown to be equally successful in evaluating cognitive fatigue as longer-administered versions [15]. Drawbacks observed from lengthier administered adaptations include an increased resource demand on participants due to the time on task required; this could potentially be harmful to participants already suffering from cognitive fatigue due to an ABI. The design of some of the PVTs mentioned above allowed for a degree of learning or preempting that occurred in these studies [20,21]. This problem needs to be addressed for tests to be equally taxing over time, so that results are continually obtained under the same level of effort. Johansson et al [18] compared the utility of the MFS to a suite of cognitive tests to determine if there is a direct correlation in their ability to subjectively and objectively measure cognitive fatigue. Neuropsychological tests that were employed included Digit Symbol Coding, Digit Span, Spatial Span [23], and Trail Making [24], which were used to measure processing speed, cognitive attention, working memory, verbal fluency, and reading speed. It was concluded that subjective cognitive fatigue following brain injury mainly correlates with objectively measured information processing speed [18]. In particular, specific cognitive tests from the Wechsler Adult Intelligence Scale, such as Spatial Span, have been shown to be effective when used to evaluate cognitive fatigue in terms of cognitive attention and working memory [8]. Consequently, these computerized tests allow for a detailed level of accuracy in testing, thus providing a more comprehensive view of cognitive ability than can be found using paper-based tests [16,18]. Work by Kay and Rector [16] and Gartenberg and McGarry [20] investigated the efficiency of using short mobile-based tests, along with the potential usability issues that arise from test delivery on a mobile platform. These studies concluded that mobile-based assessment approaches were just as effective as desktop computer-based approaches [16,20]. Due to the simple design of tests such as the PVT, there were no usability issues noted when delivering it using a mobile platform. By contrast, Swendeman et al [25] conducted a study examining the validity of using a self-assessment-based approach on a smartphone; the study aimed to evaluate emotional and behavioral self-reports daily over a 6-week period. Daily completion rates were reported to be 50%, with 70% of participants completing three follow-up surveys after the 6-week period. However, adherence to daily assessment was observed to be low, which was attributed to errors in data that subsequently had to be excluded from the evaluation results. To resolve this issue, it was suggested that prior training with the technology should be provided for participants, as it is often the case that brain-injured individuals find technology difficult to use due to their condition [26].

The study presented in this paper aimed to address the research question, “ *How can cognitive fatigue be accurately assessed through the use of a mobile assistive technology?”.* As such, the study sought to evaluate the correlations between subjective and objective measures of cognitive fatigue, with a future view to supplement traditional clinical evaluations with in situ cognitive assessment. From this approach, the following hypotheses were formulated: (1) a correlation will be observed between objective cognitive testing performance and the subjective measure reported by the MFS; (2) participants who are cognitively fatigued would exhibit a reduced level of performance in Spatial Span, PVT, and Mental Arithmetic Tests; and (3) accurate cognitive fatigue assessment can be carried out on a mobile app for use in everyday life.

## Methods

As highlighted, the assessment of cognitive fatigue commonly takes place within a clinical environment, which does not allow for accurate in situ assessment. This study primarily explored the adaptation of cognitive fatigue tests for delivery using a smartphone. During the study, the validity of the smartphone-based tests was compared to self-reported cognitive fatigue levels, as measured using the MFS. Accordingly, the MFS version employed had to be adapted to allow for delivery on a smartphone. Three cognitive tests were chosen from those identified in the literature. The Spatial Span Test was selected for inclusion to assess working memory. The PVT was also selected, as it has been proven as a cognitive fatigue evaluation method [27]; moreover, the nature of the test allows for small discrepancies in performance to be determined due to the timing of reactions being measured to approximately 1 millisecond. A Mental Arithmetic Test was also included, as simple addition and subtraction questions have been shown to successfully measure cognitive throughput [28], and are effective in assessing the characteristics associated with cognitive fatigue [19]. The adaptation and implementation of these cognitive tests are detailed later in this paper.

### Smartphone App Design Process

**Figure 1:**
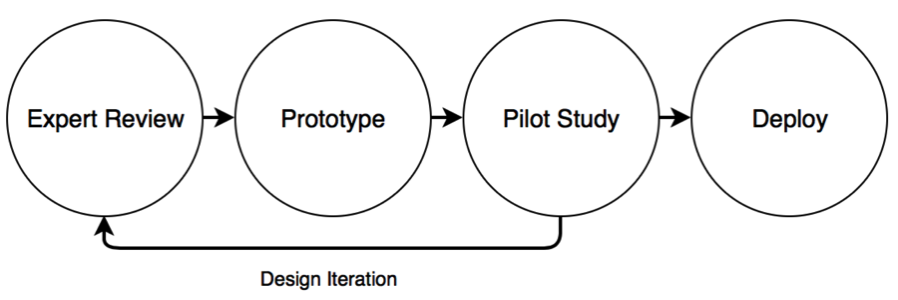
Smartphone app iterative design process.

A multi-disciplinary, iterative approach (as illustrated in Figure 1) was employed to systematically inform and evaluate each aspect of the smartphone app’s design. The research team included experts in the fields of ABI, psychology, and interaction design, which informed initial design decisions and the proposed functionality of the app. This *Expert Review* informed the development and design process to establish a *Prototype* app. In turn, this Prototype was then used in a *Pilot Study* that focused on user-centered design, to test our three hypothesized assumptions and to refine the protocol for eventual deployment during the target study. This process permitted the design and development of the app to be informed by clinical theory and practice, as well as commercial design theory and participant feedback. Table 1 shows the different development stages of the study, in addition to the requirements within each stage, that facilitated the desired outcome. Accordingly, each outcome was met before moving on to the next stage.

**Table 1:** Requirements for each Development Stage.

Development Stage	Targeted Outcome	Specific Processes	Modifications
Expert Review	Overall workflow of app finalized. Requirements list and features identified.	Research into appropriate cognitive testing methods was carried out alongside mobile workflow and design research. Expert opinion was used for each requirement inclusion.	Sequencing of cognitive tests were discussed and finalized based upon perceived enjoyment and difficulty.
Prototype	Prototype app finalized, based upon requirements set up from Expert Review.	Storyboarding of smartphone app screens to create a structured guide for design. Color scheme and visual design was finalized. Front and backend development finalized to create a stable, secure, and usable app.	Through storyboarding and visual planning, complexity of the cognitive tests was reduced to improve their usability. The Mental Fatigue Scale was adapted for easier use on a smartphone.
Pilot Study	Deployment of the prototype app to a small group of participants in a pilot study. Gather usability feedback from participants.	Carried out a two-week pilot study using the developed app. Participants did not have any prior acquired brain injuries (to validate the app on a healthy population first, as using a vulnerable population is unethical).	Feedback from the pilot study informed the team of usability issues and bugs, and participants suggested improvements to make the app more enjoyable.
Deploy	Deployment of the finalized app to a larger number of participants. Data collection to allow for analysis of results.	Targeted study was used to validate the cognitive fatigue measures that were used within the app.	Targeted study deployment allowed for data collection through participant use of the finalized app. This data was then analyzed to establish the accuracy and validity of proposed cognitive testing methods.

#### Expert Review

The initial phase of the design process primarily focused on using the research team’s multidisciplinary expertise to inform the overall nature and workflow of the app. The order in which the cognitive tests would be delivered was considered an important design decision, as retaining participant engagement (particularly when cognitively fatigued) is crucial. Through Expert Review, the order of the selected tests was based on a combination of the perceived level of difficulty and expected level of participant enjoyment. These considerations subsequently lead to potentially less-engaging tests being sequenced earlier in the workflow of the app, whereas more engaging tests were sequenced later. Accordingly, the cognitive tests presented to participants would become increasingly stimulating over the course of a session. As a result, the ordering of the tests presented by the app was: MFS, Spatial Span Test, PVT, and Mental Arithmetic Test. In addition, the Expert Review also helped to ensure that any changes made to the MFS, specifically its redesign for optimal presentation on a smartphone screen, did not compromise the validity of the corresponding results.

#### Pilot Study

Following the initial Expert Review stage, a Pilot Study was delivered to a small set of participants (n=5), using a first-iteration prototype app. This stage facilitated an evaluation of the efficacy of each of the selected cognitive tests to help ensure that they would challenge participants sufficiently, and facilitate collection of a dataset with appropriate variability to permit subsequent analyses. The Pilot Study also provided an opportunity to gain feedback on information design and visual design choices made during the Expert Review, which might affect the usability of the app.

#### Iterative Improvements to Mobile App Cognitive Test Design

The iterative nature of the design process allowed for further Expert Review to inform a response to findings from the Pilot Study, which helped to further refine and optimize the design of the app and the planned larger-scale study. A notable instance of this process is the design of the Spatial Span Test, which was revised from a 5x5 grid to a 4x4 grid layout, as the original design proved difficult to accurately tap when displayed on a smartphone screen. Additionally, a countdown timer and progress bar were added to inform participants of both the remaining time during the test and their ongoing progress.

The design of the PVT was also revised to introduce a randomly positioned stimulus, to prevent participants from preempting a response. Immediate visual feedback was also incorporated to encourage concentration and participation during the test. In terms of the Mental Arithmetic Test, initial concerns regarding the layout of the on-screen number keypad used for participant’s responses was validated, with a telephone style layout proving acceptable to participants. Moreover, changes to the MFS element of the app required much more care, as the purpose of including it was to provide an established cognitive fatigue assessment tool to help evaluate how effective the mobile cognitive tests were at assessing cognitive fatigue. To maintain the validity of the MFS, it was important that it differed as little as possible from the original paper-based questionnaire. However, the presentation of each question as a separate element marks a departure from the original paper-based version; this change was made in response to feedback from the design process that found a single continuous onscreen set of questions to be awkward from a user-interaction viewpoint.

### Target Study Delivery

**Figure 2:**
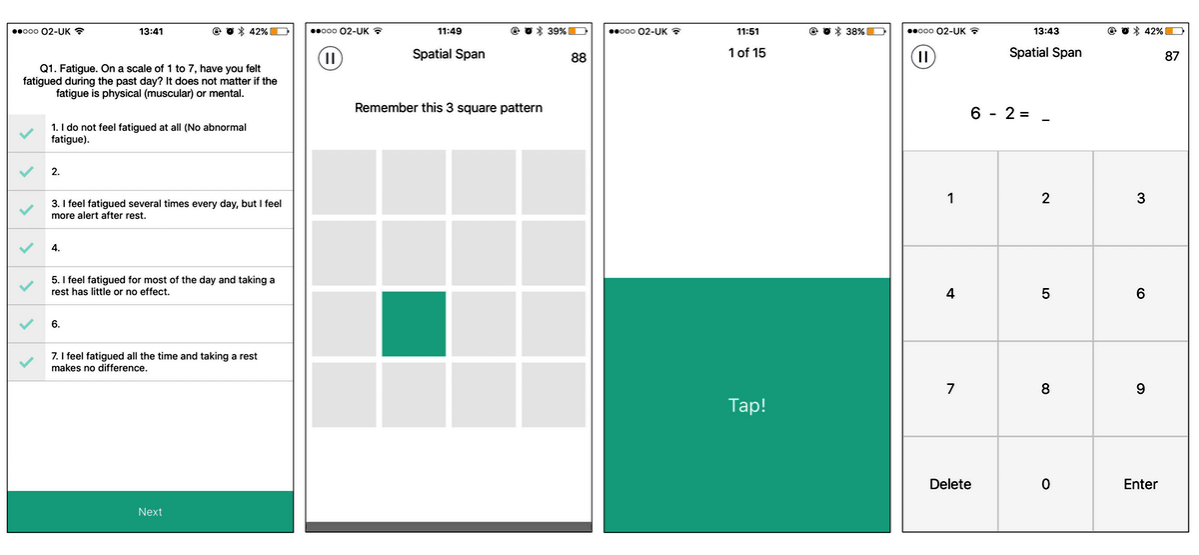
Screenshots of the smartphone app’s four main screens from left to right: Mental Fatigue Scale, Spatial Span Test, Psychomotor Vigilance Task, and Mental Arithmetic Test.

Upon completion of different iterations of Expert Review, Pilot Study, and subsequent iterations of Prototype development, the smartphone app was ready to *Deploy* within the target study. At the start of the study, guidance was given to each participant on the nature of the study and informed consent was obtained, for the purpose of conducting the study in an ethical manner. Subsequently, a brief tutorial on how to use the app was presented. Once the study had commenced, participants received a push notification daily at 3 o’clock p.m. as a reminder to carry out a session with the app. Upon launching the app, the participant was initially presented with brief instructions regarding the purpose and usage of the app, which was subsequently followed by delivery of the MFS and the set of cognitive tests in the predefined order: (1) MFS; (2) Spatial Span Test; (3) PVT; and (4) Mental Arithmetic Test. Completion of both the MFS and set of cognitive tests took approximately 5 minutes in total. Upon completion, the data collected was initially stored on the mobile device, before subsequent transmission to an online database. The set of tests presented to participants are illustrated in Figure 2. The individual tests and sequencing within the app are detailed in Textbox 1.

**Sequencing of measures within the smartphone application.**

- As shown on the left side of Figure 2, the first test presented was the MFS, which displayed 15 individual questions to participants, along with an associated 7-point response scale, each on a separate screen with a button at the bottom to progress to the next question.
- The second test presented was the Spatial Span Test, which required each participant to recreate a sequence of flashing boxes that appeared on the screen one after the other in a grid formation. If the participant correctly repeated the presented sequence, they were then presented with a new sequence that contained one additional box. Conversely, if the participant entered an incorrect sequence, then the next sequence was one box shorter, with a minimum sequence of three boxes being presented. The use of adaptive difficulty in this manner was intended to help maintain engagement regardless of the participant’s level of performance. The Spatial Span Test lasted for a total of 90 seconds, during which time participants attempted to complete as many sequences as possible.
- Upon completion, the PVT was presente: the mobile implementation of PVT required the participant to attempt to achieve the quickest reaction times possible in response to the presentation of onscreen stimuli 20 times; each time a large block of color was randomly displayed on one half of the smartphone screen.
- The final test presented was the Mental Arithmetic Test, as illustrated on the right side of Figure 2, which required participants to make a single attempt at solving a number of serial addition and subtraction tasks within a predefined time limit of 90 seconds. Although the components of the individual arithmetic operations were randomly generated, they were restricted to ensure that the problem numbers were in the range 0-9 and the result was a positive number in the range 0-18. Immediate visual feedback was provided on the correctness of the answer before presenting the next addition or subtraction task.

**Table 2:** Data collected from mobile fatigue assessment app.

Test	Factors Measured	Data Collected	Type of Data
Mental Fatigue Scale	Overall fatigue assessment	Date and time test was started	Date/time
		Questionnaire results	Numerical array
Spatial Span Test	Cognitive attention	Total number of sequences complete	Numerical
	Working memory	Longest sequence achieved	Numerical
		Total number of correct sequences	Numerical
		Total number of incorrect sequences	Numerical
		Time to complete each full sequence	Numerical array
Psychomotor Vigilance Test	Alertness	Reaction times	Numerical array
	Reaction time	Total number of premature reactions (wrong)	Numerical
		Total number of timely reactions (right)	Numerical
Mental Arithmetic Test	Cognitive throughput	Total number of correct answers	Numerical
		Total number of incorrect answers	Numerical
		Total number of questions presented	Numerical array
		Correct answer	Numerical array
		User’s answer	Numerical array

#### Data Collection

Each specific test captured a range of different data points to assess relative performance. These data included correct and incorrect responses and time it took to respond during each task. Data that was collected via the app is detailed in Table 2. All collected data were stored locally on the participant’s device before being transmitted to a secure central server.

#### Participant Feedback

Upon conclusion of the study, all participants were invited to provide feedback on the smartphone app using an online version of the System Usability Scale (SUS) [29]. For the SUS, participants were asked to score 10 items with one of five responses ranging from *Strongly Agree* to *Strongly Disagree*. Additionally, participants were asked to freely comment on any aspect of the app or study to provide further feedback.

#### Participant Recruitment

Participants (n=21) were recruited within Ulster University to undertake the study over a two-week period. Participants with no prior brain injuries were explicitly chosen, as it was considered unethical to test a newly developed method on a clinical population without first understanding how it might be of benefit. Moreover, the experience of cognitive fatigue, while a characteristic of ABI, is not limited to the condition. The mean age of participants recruited was 22 years (standard deviation=4). In addition, participants were required to own an iPhone to ensure that they were familiar with using iOS-based smartphone apps, which removed the need for additional training in device use prior to undertaking the study. Participants were required to use the app once daily to self-assess their cognitive fatigue levels. During the study, 81 individual testing instances were recorded, resulting in an overall daily adherence by participants of 28%.

### Target Study Statistical Analysis

Data were analyzed using IBM SPSS version 22. Descriptive statistics were used to provide information on average reaction time, MFS questionnaire total score, total number of timely reactions, total number of correct Spatial Span sequences, total number of correct answers to Mental Arithmetic questions, and longest sequence achieved in the Spatial Span Test (Table 3). Pearson’s correlations explored the relationships between the MFS and the three cognitive tests. Finally, multiple linear regression was utilized to investigate variables thought to be predictive of fatigue. Although prompting notifications were issued at 3 o’clock p.m. every day, participants could take the test at any time they chose. This flexibility facilitated a range of testing instances throughout the day. Data obtained from the overall set of results was grouped into three episodic epochs and analyzed separately: (1) morning (midnight to midday); (2) afternoon (midday to 6 o’clock p.m.); and (3) evening (6 o’clock p.m. to midnight). Subsequently, the groupings were utilized to assess if there was a predominant time of day when fatigue levels were higher, based on self-reported figures.

**Table 3:** Correlations of testing variables to the self-reporting MFS scale.

Mental Fatigue Scale Score	Average Psychomotor Vigilance Task Reaction Time	Average Psychomotor Vigilance Task Reactions Correct	Average Spatial Span Correct	Average Arithmetic Questions Correct	Average Highest Spatial Score Reached
Correlation	.342^a^	.159	-.141	-.016	-.064
Significance (P-value)	.008	.157	.209	.884	.568

^a^Correlation is significant at the 0.01 level (2-tailed).

#### Data Exclusion

To identify outliers of extreme and incorrect testing instances, data were analyzed using box plots. Analysis of outliers used the standard measure of 1.5 times the interquartile range to find outliers that were viewed to be too far from the central values to be reasonable. A likely cause of outliers is inaccurate self-assessment (eg, assessment scores were abnormally high or low in comparison to testing scores), in conjunction with a simplistic pattern of responses observed (ie, all responses were the lowest or highest possible choices). Removal of outlying instances of this type helped to ensure only reliably accurate tests were included during analysis.

## Results

Overall, 81 individual testing instances were recorded by the participant population, resulting in a daily adherence by participants of 28%. Daily reminder notifications resulted in a participant adherence rate of 23% within the first two hours of receiving the notification; this finding indicates that a large proportion of app usage instances occurred due to the daily reminders.

### Correlation and Regression Analysis

Initial analysis of the results obtained indicated the average reaction time during the PVT had the strongest correlation with the MFS for evaluating cognitive fatigue, measuring 0.342 (*P*=.008). By comparison, the average Spatial Span sequences correct had a correlation of -0.141 (*P*=.209), and the total Mental Arithmetic questions correct had a correlation of -0.016 (*P*=.884). All correlations are detailed in Table 4.

The best correlation to the MFS was from the average reaction times in the PVT. The significance of this correlation shows that the PVT may be a valid way to assess cognitive fatigue as a replacement for self-reporting. Average reaction time, total number of correct reactions, total number of correct Spatial Span sequences, and total number of correct Mental Arithmetic questions were entered into a multiple linear regression model to determine which factors were predictive of fatigue (see Table 5). Correspondingly, a statistically significant model emerged (*P*=.038, F=2.682), which explained 8% (adjusted R-square=.078) of the variance observed in fatigue scores. The only statistically significant variable to predict fatigue was PVT average reaction time.

**Table 4:** Correlations of testing variables against the self-reported MFS scale.

Mental Fatigue Scale Score	Average Psychomotor Vigilance Task Reaction Time		Average Psychomotor Vigilance Task Reactions Correct		Average Spatial Span Correct		Average Arithmetic Questions Correct		Average Highest Spatial Score Reached
Correlation	.342^a^		.159		-.141		-.016		-.064	
Significance (P-value)	.008		.157		.209		.884		.568	

^a^Correlation is significant at the 0.01 level (2-tailed).

**Table 5:** Standardized and unstandardized regression coefficients for variables entered into the model.

Variable	B	Standard Error	Βeta Coefficient	T-statistic	P-value	95% CI
Mental Fatigue Scale	-6.990	27.042	N/A	-0.258	.797	-60.848 to 46.869
Average Reaction Time	46.610	18.898	.312	2.466	.016	8.971 to 84.249
Average Reactions Correct	1.442	1.654	.099	0.872	.386	-1.853 to 4.737
Average Spatial Span Correct	-3.505	2.991	-.138	-1.172	.245	-9.462 to 2.452
Total Mental Arithmetic Correct	0.696	0.488	.179	1.427	.158	-.276 to 1.668

**Figure 3:**
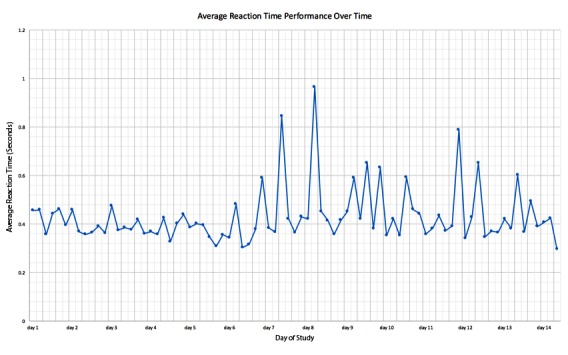
Average reaction time performance over the duration of the study.

### Time Series Trend Analysis

Self-reported cognitive fatigue measured by the MFS was also shown to correlate with a slower average reaction time, which supports the hypothesis that information processing speed is a predictor of fatigue. From Figure 3, it can be observed that there is no significant increase or decrease in performance in the PVT over the two-week testing period. Average reaction time stayed consistent apart from several outliers above the upper control limit, which could be attributed to lapses in concentration. If a significant performance increase were observed, it would suggest a level of training and learning over time; however, this is not the case from the overall results obtained.

As previously mentioned, results data were grouped into three epochs throughout the day (*morning*, *afternoon*, *evening*). Analysis of the results obtained from the MFS demonstrates that the morning epoch produced the highest self-reported levels of cognitive fatigue, which were 30.18% higher than during the afternoon epoch, and 28.21% higher than during the evening epoch. Although there was no statistical significance observed, there is an interesting correlation with the metrics outlined in Textbox 2 all being lower, which also indicates higher levels of cognitive fatigue.

It may be appropriate for future work to further analyze the time of day that tests were undertaken. Consequently, this data could support the hypothesis that a reduced level of performance would be seen in participants with higher self-reported fatigue (see Table 6).

After completion of the study, participants were further invited to evaluate the smartphone app using an online version of the SUS and to comment on changes to the app that could encourage higher levels of participation. Subsequently, results obtained from this evaluation indicated that the primary reason for nonadherence was the MFS, which was considered *boring* and *strenuous* in terms of having to answer multiple questions. By comparison, the cognitive tests were perceived to be *fun* and *enjoyable*. This finding supports the study in trying to determine an accurate and more engaging method of in situ fatigue evaluation than is currently available.

**Observed lower scores during morning epoch.**

- Mean amount of correctly answered arithmetic questions was 4.34% lower during the morning epoch than the afternoon epoch and 4.58% lower than the evening epoch
- The longest sequence achieved in the Spatial Span test was 1.05% lower in the morning epoch than the afternoon epoch and 1.57% lower than the evening epoch
- The total number of correct Spatial Span sequences achieved was 0.41% lower in the morning epoch than the afternoon epoch and 1.63% lower than the evening epoch

**Table 6:** Descriptive statistics for morning, afternoon, and evening epochs.

Epoch		N	Minimum	Maximum	Mean	Standard Deviation
**Morning**						
	Reaction time (seconds)	15	0.34846	0.60071	0.41786^a^	0.07409
	Mental Fatigue Scale result	15	24	84	40.93	17.123
	Total number of timely reactions	15	12	15	14.53	0.834
	Total number of correct Spatial Span sequences	15	4	5	4.87	0.352
	Total number of correct Mental Arithmetic answers	15	15	28	23.47	3.461
	Longest Spatial Score sequence reached	15	4	7	5.67	0.9
**Afternoon**						
	Reaction time (seconds)	45	0.2950	0.65199	0.386^a^	0.05733
	Mental Fatigue Scale result	45	14	78	30.2	12.836
	Total number of timely reactions	45	12	15	13.89	0.959
	Total number of correct Spatial Span sequences	45	4	6	4.89	0.573
	Total number of correct Mental Arithmetic answers	45	14	31	24.51	3.501
	Longest Spatial Score sequence reached	45	3	9	5.73	1.372
**Evening**						
	Reaction time (seconds)	21	0.3069	0.7600	0.37417^a^	0.1443
	Mental Fatigue Scale result	21	14	53	30.81	10.731
	Total number of timely reactions	21	12	15	14	0.894
	Total number of correct Spatial Span sequences	21	4	6	4.95	0.590
	Total number of correct Mental Arithmetic answers	21	19	31	24.57	3.682
	Longest Spatial Score sequence reached	21	3	9	5.76	1.411

^a^Denotes the median reaction time

## Discussion

While it is clear from the results obtained that all cognitive fatigue tests showed a degradation in the participant’s performance when there is a higher level of self-reported fatigue (as hypothesized prior to the study), analysis indicates that only the PVT is significant enough to make claims on its validity. Subsequently, both the Spatial Span Test and Mental Arithmetic Test do not provide a strong correlation with the MFS for measuring cognitive fatigue. Although a correlation may be observed from all three of the tests as hypothesized, currently only the PVT has the potential to accurately assess cognitive fatigue using a mobile device, as it is able to determine the current level of mental fatigue concordant with the levels reported using the MFS. Furthermore, PVT is less demanding for participants to undertake than the other cognitive tests. Feedback from a usability viewpoint also suggested that the PVT was considered by participants to be more engaging than the MFS. From a participant perspective, assessment of cognitive fatigue is feasible via a smartphone app; however, adherence to regular testing is crucial to gaining an understanding of the condition over time.

Adaptation of the smartphone app to increase the overall time on the cognitive tests could potentially lead to more accurate evaluation results across all tests, although this is only possible if participants are willing to take part in a longer daily task. Tailoring time-on-task to promote continued adherence, while also collecting sufficient data to accurately assess the condition, is a critical balance that requires further research. Such studies will enable a greater understanding of the testing duration required to permit accurate mobile-based cognitive fatigue evaluation. Future work may benefit from increased time-on-task for each of the three cognitive tests.

When considering the scores of the PVT from individual participants, it can be observed that there was no improvement in scores over time. It is important that the participants find the test equally taxing over time, so that the same indicators of fatigue are present, rather than a participant learning from previous sessions and becoming increasingly expert in undertaking the assessment. Such learning would consequentially mask any underlying cognitive fatigue.

### Principal Findings

The approach of interpreting fatigue using a mobile device has been demonstrated to have validity. However, a number of improvements could be made to the process, in addition to changes to the specific data that is collected. Such changes could include collecting data on environmental factors such as the quantity and quality of sleep, daily exercise levels, current location and social environment, and current emotional status. Based on the study performed, participant feedback indicated that carrying out the MFS was one of the least enjoyable parts of the overall process, which potentially lead to a reduction in engagement. The use of PVT has been shown to provide a similar capability in the assessment of cognitive fatigue, meaning that future work can potentially exclude the use of MFS. Subsequently, this exclusion may further increase user participation rates, which in turn may potentially increase the accuracy of the cognitive fatigue evaluation. An abridged variation of PVT was employed within the smartphone app; however, it is anticipated that a higher degree of accuracy could be achieved by using a longer test session. Accordingly, future work should increase the length of the PVT utilized, which could produce a more precise indicator of fatigue.

### Limitations

A limitation of the study is that daily participation was not enforced. This enforcement could have increased the amount of data obtained, thereby improving the confidence of the findings. Given the real-world approach employed throughout the study, the only available (and appropriate) participation encouragement mechanism was seen to be mobile device notifications. Future work will aim to improve participation rates, in conjunction with increasing the amount of data collected to potentially obtain a more accurate assessment of daily fatigue levels. Furthermore, all cognitive test metrics that were employed alongside the MFS were constrained to a relatively short timeframe, in order to aid participation rates. However, this limitation may have had an adverse impact on the effectiveness of the tests in identifying small discrepancies in performance. Other limitations of the current study include the short duration of the overall study; future work will seek to address all of these limitations.

### Comparison with Prior Work

The validity of assessing fatigue using both the Spatial Span Test and Mental Arithmetic Test has been proven to be effective by Van Dongen et al [19] and Johansson et al [18]. In both cases, longer forms of the tests were utilized; however, a static nonmobile platform was employed for test delivery. Aspects of the research carried out by Johansson et al [18] directly corresponds with the research discussed in this paper. In their comparative study on the utility of self-assessment and cognitive tests, Johansson et al found that both the questionnaires and cognitive tests produced the same results when measuring fatigue in an individual [18]. In particular, significant decreases in information processing speed were measured using the Digit Symbol Coding and Trail Making Tests [18]. Furthermore, the cognitive tests that were used proved to be quicker than filling out a self-assessment questionnaire. Accordingly, the results obtained from the research carried out in this paper concur that information processing speed, measured through cognitive tests such as PVT, correlates to subjective measurements of fatigue.

Work carried out by Thomson et al [30] demonstrates visual reaction time using a smartphone is a simple and repeatable method for objectively monitoring the effects of sedation. Visual reaction times were found to be considerably less variable than patient-assessed sedation scores and could be used to identify impending over-sedation [30]. Again, this finding shows the validity of a reaction time test as a valid measure of attention and wakefulness. Work carried out in this paper shows its adaptability and provides evidence that it can be translated for use in measuring cognitive fatigue.

However, in terms of the efficacy of mobile-based self-assessment, Swendeman et al [25] carried out a study on the validity of self-reporting via a smartphone and found relatively low compliance with daily assessment. Consequently, these findings indicated the potential unreliability of self-assessment with respect to assessment completion rates [25]. A more playful approach to consider would be the integration of game-based testing, which may potentially improve the adherence to completion, as suggested by the results obtained from the research presented herein.

One key concern with cognitive tests (such as PVT) that have been previously adapted for a mobile platform [16] is general usability, particularly for individuals with brain injuries, due to their specific cognitive impairments. Although this concern could potentially be alleviated with initial training, it does not allow for immediate assistance if issues are encountered when used outside of a clinical environment. Kay and Rector initially adapted PVT for use with a modern touchscreen device to assess reaction time as a means to evaluate fatigue in everyday life [16]. PVT requires accurate evaluation of reaction time (down to approximately 1 millisecond), making accurate input sensitivity crucial. Within the work conducted by Kay and Rector, they first evaluated different input methods including *touchdown*, *swipe,* and *touch-up,* and concluded that participants preferred *touchdown* due to it similarities to a button press [16]. The study was then carried out with 20 participants to assess everyday fatigue, and results were comparable to those gathered from the desktop computer-based implementation of PVT [16]. Kay and Rector concluded that future work in the area should aim to add situational context to the app so that assessment could be moved away from the clinical environment, thereby reducing cost and increasing contextual evaluation when needed.

### Conclusions

Previous studies have confirmed the use of the MFS and PVT as a feasible way to assess cognitive fatigue in a clinical setting. This study demonstrated that smartphone-based adaptations of these proven methods are internally compatible methods of assessing cognitive fatigue in situ. The smartphone app presented in this research provides a potentially effective tool for the individual evaluation of cognitive fatigue levels in situations where formal intervention and assessment approaches are neither feasible nor available. Furthermore, the use of smartphone app-based fatigue assessment permits evaluation to be carried out on a continual daily basis.

All three of the cognitive tests employed within the smartphone app produced positive participant feedback, with some participants even indicating that they would like personal scores, as it would further encourage them to participate more frequently. Consequently, by introducing a competitive aspect to the cognitive tests, participant effort and daily participation rates could potentially be improved. Future work may additionally permit the provision of real-time data to relevant medical professionals, so effective and timely interventions can be arranged if extreme fatigue becomes apparent. Correspondingly, there exists three main areas that in situ fatigue assessment could benefit from sensor data and contextual factors: (1) to improve notifications so that daily participation can be increased; (2) to provide additional data that may give a more precise indication of the occurrence of cognitive fatigue; and (3) to track a participant’s daily activity and advise them on appropriate steps to further combat cognitive fatigue.

Based on the three episodic epochs, results from both the MFS and PVT indicated that the morning epoch produced higher levels of fatigue. Accordingly, this knowledge could be employed on an individual basis to help tailor the time of delivery of the test session, to pinpoint higher fatigue levels throughout the day. Adaptation of notifications (based on collected data over time) would further facilitate assessment when fatigue is usually at its highest. Correspondingly, identifying the time of day when fatigue levels are high offers an increased ability to prevent mental fatigue by preempting its arrival and suggesting steps to take to reduce its severity. Assessing the time of an individual’s participation could help inform notifications for the most likely time of adherence.

## Abbreviations

ABI: Acquired Brain Injury
FSS: Fatigue Severity Scale
MFS: Mental Fatigue Scale
PVT: Psychomotor Vigilance Task
SUS: System Usability Scale
VAS-F: Visual Analogue Scale for Fatigue
